# School-based smoking prevention strategies for adolescents in a conservative LMIC context: a qualitative study from Egypt

**DOI:** 10.3389/fpubh.2025.1694729

**Published:** 2025-10-27

**Authors:** Maryam Ba-Break, Bridgette M. Bewick, Helen Elsey, Reinhard Huss, Tim Ensor, Mohamed Saleh, Sean Donnelly, Doaa Mohamed Osman

**Affiliations:** ^1^School of Medicine, University of Leeds, Leeds, United Kingdom; ^2^Department of Health Sciences, University of York, York, United Kingdom; ^3^Universal Basic Income Leeds Lab, Leeds, United Kingdom; ^4^Tameside and Glossop Integrated Care NHS Foundation Trust, Manchester, United Kingdom; ^5^Public Health and Community Medicine Department, Faculty of Medicine, Assiut University, Assiut, Egypt

**Keywords:** adolescent, smoking, prevention, tobacco control, school-based intervention, COM-B model, behaviour change, Egypt

## Abstract

**Objective:**

Adolescent smoking is a critical form of psychoactive substance misuse, particularly in low- and middle-income countries like Egypt, where youth tobacco use remains a public health concern. This study explored current and potential school-based smoking prevention interventions in a conservative low- and middle-income country context, to identify strategies which enhance adolescents’ capability, opportunity, and motivation to avoid smoking initiation.

**Methods:**

A qualitative study was conducted in three public preparatory schools (boys’, girls’, and mixed gender) in Asyut, Upper Egypt. Data was collected through 40 semi-structured interviews with school staff, 16 focus group discussions with 76 pupils (aged 12–13), and analysis of 172 school documents. Creative tools including picture-elicitation and story-making were used with pupils. Data was analysed using the framework approach, guided by the Capability-Opportunity-Motivation-Behaviour model.

**Results:**

Seven meta-themes were identified. Key strategies included educating pupils on the broad consequences of smoking, including health, appearance, fitness, finances, relationships, addiction, religious values, and equipping them with refusal and coping skills. Interventions should promote smoke-free school and home environments, model non-smoking behaviour, and leverage peer influence. Motivational strategies such as storytelling, real-life examples, reward schemes, and accessible extracurricular alternatives were also emphasised. Barriers included limited resources, cultural taboos, and misinformation around e-cigarettes.

**Conclusion:**

School-based smoking prevention interventions in low- and middle-income country settings must be proactive rather than reactive, multi-dimensional and culturally appropriate. Applying the Capability-Opportunity-Motivation-Behaviour model can help schools design integrated interventions that build psychological capability, reshape opportunities, and strengthen both reflective and automatic motivation in early adolescence. The findings have direct implications for public health practice, school policy, and substance misuse prevention programming in resource-constrained and socially conservative settings.

## Introduction

Tobacco use remains a global epidemic as 22% of adults smoke, with this rate increasing annually (1). Smoking is a major risk factor for Non-Communicable Diseases (NCD) which account for 70% of global deaths (2). Smoking-related morbidity and mortality reduce labour productivity and income-earning potential, alongside disrupting economic growth (3). Smoking prevention is therefore a global priority, with large-scale international studies identifying adolescents smoking increases the risk of life-long and heavier smoking behaviours (4, 5), hence why roughly half a trillion US dollars is spent annually on global tobacco control measures (6).

Egypt holds the highest tobacco consumption in the Arab world and the second highest in the Eastern Mediterranean region (MENA), as 23.8 and 19.7% of adults have or currently use tobacco, respectively (7, 8). Smoked tobacco remains most popular among current tobacco users with 84% smoking manufactured cigarettes and 17% smoking Water-pipe (8). 95% of smokers reportedly smoke daily, of which 80% smoke 16–20 cigarettes and 25% smoke within 5 mins of awakening (Nicotine dependants) (8). Scarce e-cigarette usage data exists for Egypt, with 18% of respondents in one study having trialled e-cigarettes, nearly half of which reported daily use (9). Concerningly, e-cigarette use prevalence is rising among youths/students and never smokers (10). Over the previous 30 years, smoking prevalence has increased four times faster than population growth, 8–9% vs. 2% (11–13). Egypt has the cheapest manufactured cigarettes in the MENA (14), although they became less affordable after a 40% tax increment in 2014 (3). A 7.3% increase in illicit tobacco trade over 3 years created greater accessibility to affordable cigarettes, further fuelling smoking among the poor population (15).

Adolescent smoking in Egypt presents another significant public health problem, as 31.3% of 13–15 aged pupils have used tobacco and 13.6% are current tobacco users (12, 16). Gender-based adolescent smoking prevalence variations also exist, with prevalences between 15.4–33.1% for males and 3.3–10.65% for females (17, 18). As nicotine is a psychoactive substance, adolescent tobacco use represents an important form of substance misuse, often co-occurring with other risk behaviours (19). The national average age to initiate daily smoking among adults is 16 years old (8), however this rises to 20 years old among adolescents aged 13–15 who have ever smoked. 89.4% tried a cigarette before 14 years old (12, 20) and 32.3% tried flavoured Water-pipe (Shisha) before 11 years old (16). Concerning, current cigarette smokers aged 13–15 are smoking heavily and frequently, with 62% nicotine dependents and 74% smoking every 4 h (21). While 52.4% of current Shisha smokers aged 13–15 smoke at least two sessions daily (16).

Without effective interventions, high adolescent smoking rates will result in a heavy public health burden for a country as large and young as Egypt – with 60% of its 91.51 million people aged below 24 years (22). Smoking prevention interventions should therefore target adolescents, since smoker adolescents are more likely to be nicotine dependent, substance abusers (19) and regular smoker adults (7, 23). Additionally, smoking delays adolescent height and weight growth (24–26) and increases the risk of physical and psychological complications of NCD earlier in life (27–30).

Prevention of smoking initiation is prioritised in the WHO global agenda for tobacco epidemic control (31), which aimed to accelerate tobacco use reduction by guiding countries’ implementation of the WHO Framework Convention on Tobacco Control (FCTC). Egypt signed the FCTC in 2003 (32), and developed national tobacco control policies aligning with it (33) aiming to reduce current tobacco use rates 30% by 2025 among those aged 15 years (34). Preventing adolescents from smoking initiation is crucial in achieving this, as quitting smoking is challenging once habitualised and the probability of quitting is inversely proportional to initiation age (35, 36).

Over the past three decades, many countries have used school-based interventions to prevent adolescents smoking (37) as this environment offers efficient and equitable access to adolescents, where behavioural change activities can be integrated into school curriculums (38–42). High school attendance rates among Egyptian adolescents make schools ideal locations for targeting adolescents smoking prevention (43, 44). However, limited evidence is available on current School-based Smoking Prevention Interventions (SBSPIs) in Egypt. In LMICs like Egypt, where health systems face resource constraints and cultural influences shape behaviour, school-based interventions must be contextually adapted to ensure relevance and impact (45). A key step in designing culturally sensitive interventions is understanding current interventions and what other appropriate, affordable activities could be implemented (46, 47). Inadequate understanding of such issues adversely affects interventions acceptability and sustainability despite similar interventions’ success in another context (48–50).

This study aimed to identify the current and potential strategies for SBPSIs to prevent adolescents from smoking in Egypt.

## Methods

A pragmatic qualitative approach (51, 52) was employed to explore SBSPIs in three preparatory schools (boys’, girls’, and mixed gender) in Asyut City, Upper Egypt. This included document reviews, semi-structured interviews with staff, and focus group discussions (FGDs) and written essays with pupils.

### Study site and school selection

Upper Egypt was selected due to its high rates of adolescent and adult smoking, early smoking initiation and socioeconomic challenges, especially in Asyut, the Egyptian governorate with the highest poverty rate and a demographic profile similar to national averages (15, 53, 54). The presence of significant Muslim and Christian populations also allowed exploration of religious influences on smoking (55). Preparatory schools were selected because most adolescents initiate smoking before age 14, making pupils aged 12–13 ideal targets for prevention (15). To account for gender-specific smoking patterns, one school of each type (boys’, girls’, and mixed-gender) was randomly chosen from the public system, which provides free education to 92% of Egyptian pupils (44, 54). Schools were first stratified by type, and one school was randomly selected within each stratum in consultation with local education authorities, based on access and willingness to participate.

### Participant recruitment

Forty staff members were interviewed, and 16 FGDs (eight for each gender) were conducted with 76 pupils. Staff were purposefully selected for their roles in smoking prevention (e.g., headteachers, deputies, social workers, psychologists, science and religion teachers), with further participants identified through staff and pupil recommendations. FGDs included boys and girls separately, respecting cultural norms and minimising peer pressure. Participants were chosen to reflect diversity in academic performance, social engagement, and exposure to smoking, based on teacher input to reduce bias as recommended while researching adolescent smoking (56–59). Participants’ homogeneity in each FGD was protected to minimise dominance during discussions (56, 60).

### Data collection

A pragmatic approach employing multiple qualitative methods was used to gain rich, triangulated insights (52). Semi-structured interviews with staff explored perceptions and experiences of smoking prevention and were guided by flexible prompts encouraging open discussion. FGDs were carefully designed to minimise dominance and peer pressure, build trust, and maintain cultural sensitivity. Pupils engaged in FGDs enhanced by creative techniques—including writing on cards, picture-elicitation, and story-making—to support age-appropriate, culturally sensitive discussions. Each FGD involved 4–5 participants to ensure engagement. As recommended while researching young people (61–63), story-making was utilised to allow group collaboration and individual expression, enabling deeper exploration of pupils’ perspectives. Document review was conducted throughout the study using Miller and Alvarado (64) framework, analysing 172 school documents (e.g., curricula, policy manuals) for content, accessibility, and relevance to anti-smoking efforts. This provided objective, context-rich data and helped assess alignment between documented policy and practice.

Ethical approval and research clearance were obtained from relevant committees in Egypt and the United Kingdom, and informed consent procedures were followed in accordance with international ethical guidelines. Full details of the ethical approvals and consent processes are provided in the Ethics section below.

### Data analysis

Data was analysed using the framework approach, combining inductive and deductive strategies to generate local insights while aligning findings with behavioural models like the Capability Opportunity Motivation-Behaviour model (COM-B) (65). Differences by school, gender, or respondent type were noted where relevant. MAXQDA software was used in the analysis following seven iterative stages: data transcription, familiarisation, coding, developing and applying an analytical framework, matrix charting, pattern mapping and interpretation. This ensured rigorous comparison across data sources, while researcher reflections, field notes, and digital memos supported validity and cultural relevance (66).

## Results

Data saturation was reached after conducting 40 semi-structured interviews with school staff and 16 FGDs with 76 students. See Table 1 for participants characteristics. The interviewed staff included head-teachers, teachers, social workers, psychologists, and extracurricular activity leaders from boys’, girls’, and mixed-gender schools. Most were aged 30–50 years, had over 10 years of experience, and the majority reported never smoking, though five were current smokers and others had experimented or quit.

**Table 1:** School-staff participants characteristics.

The school staff	Boys		Girls		Mixed-gender		Total		
	M^1^	F	M	F	M	F	M	F	T
Head-teachers	1	0	0	0	1	0	2	0	2
Head-teachers deputies	0	0	0	1	1	0	1	1	2
Social workers	1	1	0	2	1	1	2	4	6
Psychologists	1	0	0	1	1	2	2	3	5
Teach 1^st^ & 2^nd^ preparatory grades	3	3	4	7	2	6	10	15	25
Teachers of sciences	1	1	2	1	0	1	3	3	6
Teachers of religion (Islam)	0	1	0	1	0	1	1	2	3
Teachers of religion (Christianity)	0	1	0	1	0	1	0	3	3
Popular^2^ teachers of other topics	2	0	2	4	2	3	6	7	13
Leaders of school-activities (total)	7	5	1	7	4	7	12	19	31
Sports	2	0	0	0	1	0	3	0	3
Media and publications	0	1		1	0	1	0	3	3
Health education inside schools	1	1	2	0	0	2	3	3	6
Social-media organiser	1	0	0	1	1	0	2	1	3
Theatre /roleplay	1	0	0	1	0	1	1	2	3
Vocational training	0	1	0	1	1	1	2	2	4
Smoking Control Group	1	0	0	1	1	0	2	1	3
Library-related activities/Librarian	1	0	0	2	0	1	0	3	3
Age group (years)									
<30	0	0	0	2	0	0	0	2	2
30–50	6	3	0	7	3	6	9	16	25
>50	1	1	2	0	2	3	5	4	9
Years of experience in preparatory schools									
< 5	0	0	0	1	0	1	0	2	2
5–10	1	1	0	2	1	3	2	6	8
11–20	3	2	0	2	1	2	4	6	10
> 20	3	1	2	4	3	3	8	8	16
Self-reported smoking status									
Never-smoked	2	3	2	6	2	6	6	15	21
Current smoker^3^	3	0	0	0	2	0	5	0	5
Quitted smoking	1	0	0	0	1	0	2	0	2
Only experimented smoking	1	0	0	0	0	1	1	1	2
Unreported	0	1	0	3	0	2	0	6	6
Total staff in each school	7	4	2	9	5	9	14	22	36
	11		11		14		36		
Organisers of health education activities across schools^4^	4						1	3	4
Total interviewed staff	40						15	25	40

1 M, males; F, females and T, total.

2 Nominated by pupils during FGDs.

3 Based on the GYTS definition, this include regular and occasional smokers.

4 All aged 48–54 years with at least 20 years of experiences in teaching or leading activities at preparatory schools and never smoked, except one smokes heavily.

### Current school strategies to prevent adolescents from smoking in Egypt

This section presents findings on current SBSPIs in preparatory schools in Upper Egypt. Six meta-themes were developed from 16 themes identified through analysis of data from staff, pupils, and school documents across three schools. These meta-themes reflect how existing strategies target pupils’ capability, opportunity, and motivation to avoid smoking. While a range of formal (e.g., school-ground smoking bans, school curriculum, external educators) and informal efforts (e.g., posters, peer influence) exist, they are often limited in scope, inconsistently applied, and lack sustainability.

### Psychological capability: education about smoking

Pupils receive minimal, fragmented information about smoking harms through the school curriculum. Content is brief, sporadically scattered across subjects, and often outdated or optional. Lessons generally overlook harmful tobacco types such as Shisha and e-cigarettes. Although religious education sometimes addresses substance misuse including tobacco/nicotine, coverage is inconsistent and depends on pupils’ religious background. Extracurricular education on smoking harms is rare, unplanned, and typically limited to small groups selected by staff, often after pupils have exhibited smoking behaviours. External experts deliver these sessions in a didactic style with little opportunity for pupil engagement or questions. Most pupils rely on incomplete or misunderstood information from textbooks, especially when material is delivered in English without adequate language support. Visual learning materials are limited to a few old, poorly maintained posters placed in inaccessible locations. Plays and theatrical content, though effective when used, are largely reserved for competitions and not shown to the general pupil population.

### Psychological capability: skills training

Nationally developed training manuals that outline skills such as problem-solving, refusal, and stress management are available online but are not known or used by most school staff. Only a few girls in one school received such training through an externally facilitated course. Staff are willing to teach these skills but lack the resources, time, and institutional support to do so.

### Physical opportunity

All schools enforce bans on pupil smoking or bringing cigarettes to school. Random checks of pupils’ bags, clothes, and school spaces such as toilets are carried out to prevent smoking on school grounds. These measures, combined with disciplinary policies, reduce pupils’ physical opportunities to smoke at school.

### Social opportunity

Creating an anti-smoking school culture remains challenging. While teachers’ smoking on school grounds is technically banned, enforcement is weak, particularly among male teachers in boys’ schools. Teachers and visitors sometimes smoke openly, sending mixed messages to pupils. Peer influence is rarely harnessed formally for prevention, though pupils often act independently to discourage peers from smoking. School Smoking Control Groups exist but are mostly inactive or poorly resourced. Girls’ smoking incidents are kept confidential to protect the school’s reputation and preserve the social norm that girls do not smoke.

### Reflective motivation

Pupils and parents in some schools are required to sign behavioural commitment forms at the time of school enrolment. These emphasise non-smoking and are used to reinforce expectations. Pupils are regularly reminded of the consequences of smoking, including suspension or expulsion. In boys’ schools, names of students caught smoking are publicly announced, creating fear and stigma to deter others.

### Automatic motivation

Schools attempt to invoke negative emotional responses to deter smoking. Punishments—including suspension and public announcements—are applied swiftly. Informal social and mild physical punishments by trusted teachers, especially for smoking outside school, also serve as powerful deterrents. These practices, however, raise ethical concerns and are inconsistently applied.

In sum, while schools in Egypt make efforts to prevent smoking among adolescents, these strategies are limited in coverage and sustainability. Prevention efforts tend to be reactive, focus on discipline rather than health promotion, and lack integration into everyday school life. A shift toward structured, inclusive, and positively framed SBSPIs is urgently needed to achieve meaningful impact.

### Potential school-based strategies to prevent adolescents from smoking in Egypt

Following an iterative process of coding, 13 themes emerged from the data. These themes were synthesised into seven meta-themes that explain what intervention functions could be used in preparatory schools to enhance pupils’ capability, opportunity and motivation to avoid smoking, see Table 2.

**Table 2:** The coding frame on the anticipated SBSPIs in preparatory schools, Egypt.

Emerging themes	Meta themes (Intervention functions)	The COM_B components
Educating pupils about various smoking consequences	Providing knowledge about smoking consequences.	Enhancing pupils’ psychological capability to avoid smoking initiation.
Specifying harms of smoking each tobacco product		
Improving pupils’ accessibility to smoking-related information		
Enhancing pupils’ skills to avoid smoking initiation.	Provide training on skills needed to avoid smoking initiation.	
Continue implementing the current ban of smoking or carrying of cigarettes by pupils inside schools	Continue banning smoking or carrying of cigarettes by pupils inside schools	Reduce pupils’ physical opportunity to smoke at schools
Minimising pupils’ exposure to smoking by staff inside school premises	Creating a smoke-free environment and anti-smoking social norm at schools.	Reduce social opportunities at schools that enhance pupils’ smoking.
Encourage modelling of non-smokers		
Use the influence of peers to prevent smoking and create non-smoking social norm		
Stimulate thinking of the pros and cons of smoking and non-smoking.	Persuasion toward reasoning and making a self-conscious decision not to smoke.	Enhancing pupils’ reflective motivation to avoid smoking.
Use persuasive and appealing methods to raise pupils’ awareness of smoking consequences and correct pupils’ false positive beliefs about smoking.		
Creating expectations of social punishments when pupils smoke.	Negative reinforcement to enhance pupils’ decision to avoid smoking.	
Rewarding pupils who never smoked or supported non-smoking	Feeling anticipated rewards associated with non-smoking.	Enhancing pupils’ automatic motivation to avoid smoking.
Encourage reducing negative emotions	Encouraging and facilitating alternatives to smoking behaviour.	
Encouraging good use of free time		

### Psychological capability: building awareness of smoking consequences

Participants highlighted the need for culturally relevant, age-appropriate education to improve pupils’ understanding of smoking harms. Effective content should go beyond abstract health risks to include addiction, social acceptance, appearance, fitness, financial consequences, environmental harm, and religious values.

Visual and interactive materials—such as lab demonstrations, real-life stories, and scenario-based plays—were seen as especially engaging. Staff stressed the importance of correcting widespread misconceptions about the relative safety of flavoured cigarettes, Shisha, and e-cigarettes. Religion was viewed as a useful motivator, particularly among girls and more devout pupils, though it was not considered sufficient independently.

Educational delivery was often described as fragmented or outdated. Many staff expressed a willingness to integrate smoking education into the curriculum if given adequate training and physical resources, while increasing the utilisation of online national training manuals.

*“If pupils learn smoking affects appearance and fitness, they’ll avoid it…and with training, we as teachers can deliver this message better than outsiders.” (Female science teacher, boys’ school).*

### Psychological capability: training skills to avoid smoking

In addition to increasing awareness, participants emphasised the importance of equipping pupils with practical life skills to strengthen their psychological capability to resist smoking. Four core areas emerged as essential: refusal skills, self-assertiveness, stress and emotion management, and decision-making.

Refusal skills were consistently highlighted by most staff and pupils as crucial in helping adolescents navigate peer pressure. Pupils frequently reported difficulty saying no to cigarettes without jeopardising friendships or social belonging. Staff observed that many pupils accept cigarettes passively, not out of desire, but due to a lack of assertive communication skills. These findings suggest a gap between knowledge and action, underscoring the need for behavioural rehearsal and confidence-building exercises.

*“Some pupils smoke just to keep friends—teach us how to say no without losing them.” (Female psychologist, mixed-gender school).*

Self-assertiveness was described as a protective factor, particularly when pupils had strong personal goals. Boys often linked non-smoking to aspirations of maturity and self-respect, while girls associated it with future roles such as motherhood. These reflections suggest that reinforcing identity-based motivations may be a promising strategy for prevention, especially if aligned with gender-specific aspirations.

*“If you have goals… you will not smoke just for stubbornness or to prove maturity.” (Boy, boys’ school)*

*“Girls who want to be good mothers will not smoke just to prove they are equal to boys.” (Girl, mixed-gender school).*

Stress and emotion management emerged as a major concern. Both pupils and staff reported that adolescents often resort to smoking as a coping mechanism for emotional distress, whether due to academic pressure, conflict at home, or social exclusion. Many pupils lacked alternative strategies to regulate their emotions. Staff suggested that exercise, creative activities, or structured relaxation techniques could be introduced to help redirect emotional responses.

*“If pupils knew healthy ways to relieve stress, like exercise, they would not turn to smoking.” (Male head-teacher, boys’ school).*

Problem-solving and decision-making skills were also seen as underdeveloped. Pupils reported limited ability to weigh consequences or resolve conflict without external intervention. In some cases, they described turning to smoking as a way to escape problems or assert control in uncertain situations. Participants called for more opportunities to practise structured decision-making and to develop agency over personal health choices.

*“Many pupils smoke to escape problems…they need help solving them and confidence to make their own decisions.” (Girl, mixed-gender school)*

Overall, the integration of skills-based training into SBSPIs was widely regarded as a missing but vital component. In a setting where formal life-skills education is not embedded in the curriculum, participants stressed the need for dedicated, practical sessions led by trained staff. They also recommended that these sessions be interactive, scenario-based, and grounded in pupils’ everyday experiences to ensure relevance and uptake.

### Physical opportunities: maintaining the school smoking ban

Maintaining a strict smoking ban within school premises was consistently identified as a key intervention to limit pupils’ physical opportunity to access or use tobacco. In a context where supervision in homes and public spaces may be limited, schools were described as one of the few environments with enforceable and clearly defined boundaries.

Pupils understood and respected these boundaries, acknowledging that the school setting curbed their ability to carry or use cigarettes, even among those inclined to try smoking. The perceived threat of disciplinary action, combined with routine surveillance (e.g., random bag checks), served as effective deterrents.

*“Sometimes classmates speak about smoking… but we can’t smoke or bring cigarettes to school… You can only smoke in your dreams! If schools allow smoking, I will be the first one to smoke.” (Boy, boys’ school)*

However, participants recognised that limiting access to cigarettes outside school was far more challenging. Many staff felt that the school’s authority ended at its gates, and regulating tobacco sales in surrounding areas was beyond their jurisdiction. Initial progress to combat this issue has begun, as five governorates launched the pilot initiative ‘150 Metres of Safety’ in January 2025 to prevent tobacco sales within 150 metres of schools (67).

*“We can control what happens inside school, but outside—shops, street sellers—we have no power there.” (Male school manager, boys’ school)*

Despite this, some participants raised concerns about the proximity of tobacco vendors to schools, arguing that easy availability undermines internal policies and exposes pupils to temptation. Suggestions included collaborating with local authorities or community leaders to discourage cigarette sales near school entrances.

*“Shops next to schools shouldn’t sell cigarettes… It makes it too easy for boys to buy.” (Female science teacher, mixed-gender school)*

Although few participants explicitly called for formal regulation of vendors, there was general agreement that addressing environmental access outside school could enhance the impact of school-based efforts. This was seen as a potentially fruitful area for policy development and future programme expansion.

*“Even if we teach them not to smoke, they can still get cigarettes from outside… If that stops, it will support our efforts.” (Female social worker, girls’ school)*

In summary, while current school policies effectively reduce pupils’ access to cigarettes on campus, broader measures are needed to reinforce these efforts beyond school grounds. Strengthening intersectoral collaboration, particularly with local authorities and community stakeholders, may help reduce pupils’ physical opportunity to initiate smoking and improve the sustainability of prevention programmes.

### Social opportunities: creating anti-smoking norms and environments

**Figure 1:**
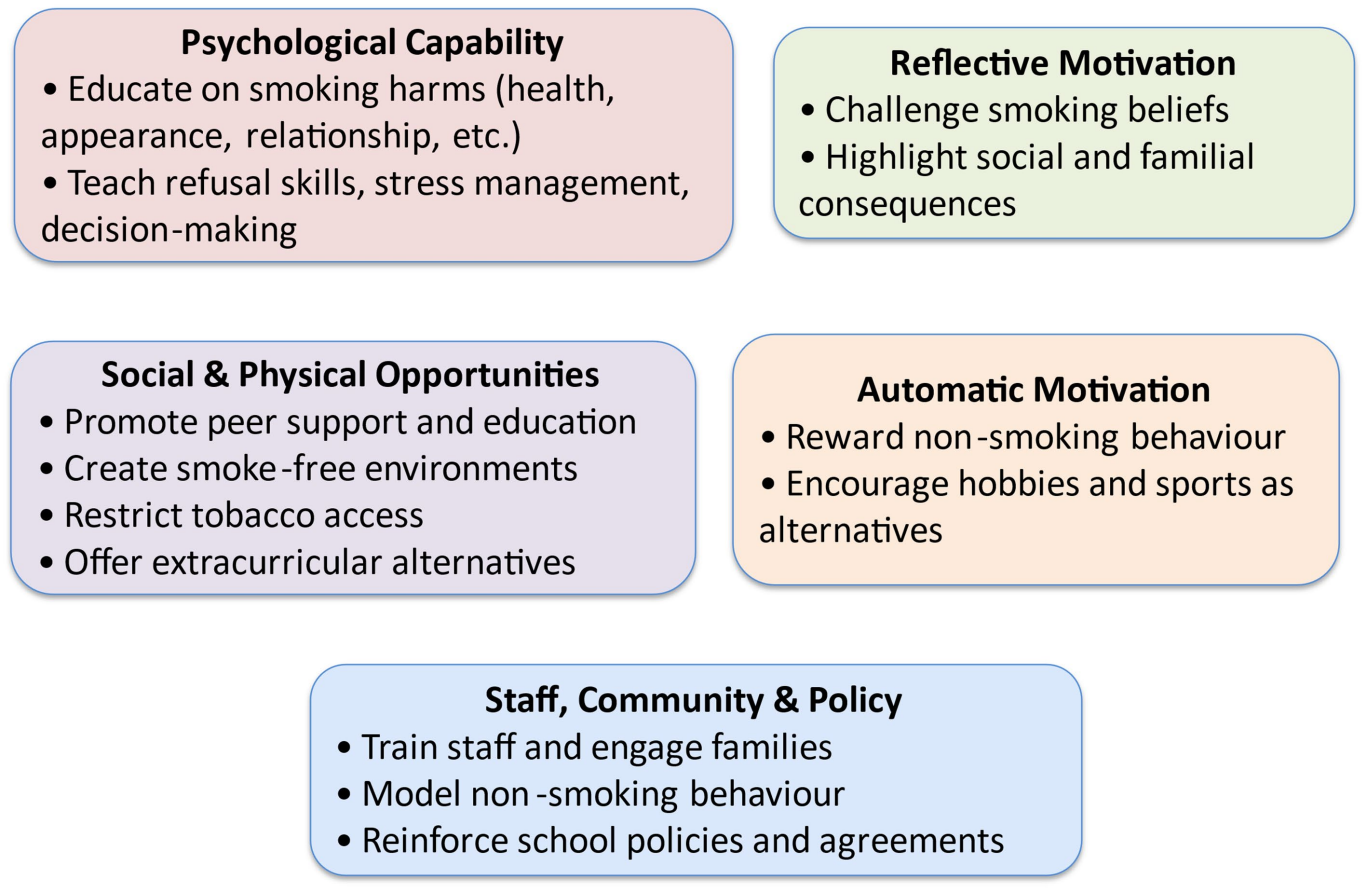
Conceptual framework of school-based smoking prevention strategies in Egypt.

Participants identified a range of strategies to reduce adolescents’ social opportunities to smoke, centred around three key areas: banning staff smoking in schools, promoting non-smoking role models, and harnessing positive peer influence. Together, these approaches aim to foster a school culture in which smoking is socially discouraged and non-smoking is normalised (Figure 1).

Staff modelling and smoke-free enforcement were seen as critical to establishing consistent anti-smoking norms. Pupils consistently described teachers as influential figures whose behaviour could either reinforce or contradict school messaging. When pupils observed staff smoking on school grounds, it created perceptions of hypocrisy and undermined the legitimacy of anti-smoking rules.

*“Teachers shouldn’t let pupils see them smoking… pupils see them as models in everything—even in smoking.” (Male science teacher, boys’ school)*

*“Teachers are allowed to smoke in school, but pupils aren’t. That’s unfair—it should be for everyone.” (Boy, mixed-gender school)*

Staff acknowledged this concern and expressed a willingness to quit if support were available. Participants called for increased awareness among school personnel about their role in modelling healthy behaviours, and many suggested offering cessation programmes for staff.

*“I know smoking is wrong. I want to quit but need help… If schools offer services, I’d stop tomorrow.” (Male sports teacher, boys’ school)*

Both pupils and staff recommended clearer, consistently enforced sanctions for teachers who smoke on school premises. Financial penalties were seen as insufficient deterrents; stronger disciplinary measures, such as warnings or suspensions, were proposed.

*“Money fines are not enough, suspensions or other penalties might be more effective.” (Male psychologist, boys’ school)*

Promoting non-smoking as aspirational behaviour was another recurring theme. Participants described the value of real-life stories, particularly when shared by admired teachers or public figures who had chosen not to smoke. These narratives were perceived as both relatable and inspiring.

*“Since I heard my teacher’s story, I decided with my friends never to smoke.” (Boy, mixed-gender school)*

*“Every teen imitates someone. Choose non-smokers and encourage us to be like them.” (Female religion teacher, boys’ school)*

While some pupils proposed publicly recognising non-smokers, concerns were raised about the potential for stigma or exclusion. Instead, integrating non-smoking into school awards or recognition systems was seen as a more inclusive approach.

*“Linking non-smoking to ideal student awards may motivate pupils, but naming individuals could alienate others.” (Female teachers, girls’ and mixed-gender schools)*

Peer influence emerged as a powerful and underutilised resource. Participants reported that informal, peer-led education, delivered by socially respected pupils, was more effective than top-down classroom lectures. Trusted peers could serve as messengers, particularly in informal spaces such as playgrounds or break time.

*“They won’t listen in class, but during breaks they will—just give me materials to talk to them about smoking.” (Boy, mixed-gender school)*

Positive peer pressure also played a key role in discouraging smoking. Pupils described how close friends could influence behaviour by offering disapproval, emotional support, or even social consequences for smoking.

*“Good friends say, ‘If you smoke, I’ll leave you’… That helps you stop.” (Boy, boys’ school)*

Teachers suggested formally recruiting socially influential pupils to champion smoke-free norms, similar to how peer leaders are used in other behavioural programmes.

*“We already use popular pupils to change bad behaviour—why not for smoking too?” (Female social worker, mixed-gender school)*

Finally, supporting pupils under peer pressure was considered essential. Respondents suggested forming anti-smoking committees composed of trained, non-smoking pupils and staff who could offer support and foster social belonging for those resisting smoking.

*“Supportive non-smoking groups can help pupils resist peer pressure and feel less isolated when they say no to smoking.”(Female social worker, girls’ school)*

In summary, creating anti-smoking norms requires a whole-school approach, where staff model healthy behaviours, non-smoking is positively reinforced, and peer influence is leveraged to cultivate a socially supportive environment.

### Reflective motivation: enhancing reasoning and self-determined non-smoking decisions

Participants consistently emphasised the importance of strengthening pupils’ reflective motivation—their capacity to rationalise and commit to smoke-free decisions through conscious reasoning. Three key strategies were identified: fostering pros/cons reflection, using emotionally persuasive educational approaches, and reinforcing social disapproval.

Encouraging critical thinking about both the benefits and risks of smoking was viewed as a foundational tool to enhance pupils’ self-regulation. Rather than focusing exclusively on health harms, participants recommended engaging adolescents in balanced discussions about what is gained or lost through smoking and non-smoking. This reflective process was seen as empowering, allowing pupils to draw their own conclusions based on values, consequences, and long-term goals.

*“Explaining harms isn’t enough…..pupils need to weigh the pros and cons to see smoking makes no sense.” (Boy, boys’ school)*

Staff also highlighted the value of real-world examples to illustrate outcomes, encouraging pupils to observe the lives of adult smokers in their communities. Personal accounts of poverty, addiction, or illness linked to smoking made risks more tangible.

*“My dad spent all our money on cigarettes—we starved. If pupils saw how smoking harms families, they’d never start” (Female vocational teacher, mixed-gender school)*

Emotionally resonant educational methods, such as storytelling, real-life case studies, and visual materials, were consistently preferred over didactic lectures. Participants described these approaches as more impactful, especially when stories were relatable and featured adolescents who began smoking early.

*“My dad told me how his friend started smoking and ruined his life—that story made me decide never to smoke.” (Boy, mixed-gender school)*

More immersive techniques, such as field visits to meet patients or families affected by tobacco-related deaths, were suggested as powerful but logistically difficult interventions.

*“If pupils met children orphaned by smoking, it would shock them and stop many from even trying it.” (Female psychologist, mixed-gender school)*

Demonstrations and visual learning tools were praised for their emotional and cognitive impact. Laboratory experiments, before-and-after posters, and health-focused imagery, particularly those showing effects on lungs, teeth, and fitness, were viewed as effective deterrents.

*“Seeing a smoker’s black lung or posters showing smoking’s effects like bad teeth or poor fitness…can scare pupils away from smoking.” (Boy, mixed-gender school)*

Videos and school plays also emerged as preferred mediums for delivering culturally relevant, age-appropriate messages. When adapted to local dialects and relatable scenarios, they stimulated empathy and peer dialogue.

*“Videos and plays that show real-life consequences of smoking help pupils relate emotionally and learn from others’ mistakes…. enough to discourage them from smoking.” (Girl, girls’ school)*

Facilitated discussions led by trusted school staff were recommended to further reinforce reasoning. These open conversations were considered more effective than authoritarian messaging, encouraging pupils to arrive at decisions independently.

*“Don’t lecture them… discuss and let them reach the right conclusion instead of telling them what to do.” (Male science teacher, girls’ school)*

Finally, anticipated social consequences, such as feelings of family shame, loss of trust, or fear of punishment, were cited as strong deterrents for both boys and girls. These socio-cultural pressures often influenced behaviour more effectively than health-based messaging alone.

*“Many pupils avoid smoking not out of conviction, but from fear of punishment and family shame … this fear alone can be a strong deterrent.” (Girl, girls’ school)*

In summary, enhancing reflective motivation requires more than conveying facts, it involves facilitating reasoning, emotional connection, and social awareness. Culturally grounded, interactive tools and trusted facilitators are essential for guiding adolescents toward self-determined, smoke-free choices.

### Automatic motivation: rewards and alternatives to discourage smoking

In addition to rational decision-making, participants emphasised the role of automatic motivation—behaviours shaped by habitual cues, emotional responses, and reinforcement—in influencing pupils’ smoking choices. Two core strategies were identified to strengthen pupils’ automatic resistance to smoking: rewarding non-smoking behaviour and offering accessible, engaging alternatives to tobacco use.

Positive reinforcement was viewed as an effective motivational tool. While punishment was acknowledged as a deterrent for some pupils, many participants believed that anticipated rewards were more influential in encouraging sustained non-smoking behaviour, particularly for pupils who may not respond to threats or sanctions.

*“Punishing smokers makes some afraid to smoke, but rewards work better with those who don’t fear penalties… it’s better to have both.” (Boy, boys’ school)*

Staff and pupils suggested a range of reward-based incentives, including small financial rewards, certificates, or public recognition, such as announcing the names of non-smokers during school assemblies or displaying them on posters. These strategies were thought to both affirm existing behaviour and inspire peers to adopt similar habits.

*“Recognising pupils who never smoked—through rewards or public praise—can motivate others to stay smoke-free.” (Female media specialist, boys’ school)*

Beyond rewarding abstinence, participants also called for recognition of pupils who actively support anti-smoking efforts among their peers. This not only reinforces prosocial behaviour but also strengthens peer norms against smoking.

*“Even small rewards for supporting peers not to smoke can boost pride and inspire others.” (Female librarian, girls’ school)*

Offering structured alternatives to smoking was widely emphasised as a critical strategy for managing emotional triggers such as boredom, stress, or peer influence. Participants noted that smoking often serves as a coping mechanism or social activity in the absence of other fulfilling options. Therefore, keeping pupils mentally and physically engaged was seen as key to reducing impulsive tobacco use.

*“To draw pupils’ attention away from smoking, they should be busy, exercises, cooking, fixing things… anything useful that fills their time.” (Boy, mixed-gender school)*

Sports and physical activity were especially valued for their stress-relieving and confidence-building benefits. In addition to promoting health, participation in sports created a sense of belonging and achievement, which could displace the emotional appeal of smoking.

*“If pupils exercise, they’ll never think of smoking… It burns stress and gives confidence.” (Male psychologist, mixed-gender school)*

However, limited access to extracurricular resources was a recurrent barrier. Many pupils, especially from low-income households, were unable to afford club memberships or materials for after-school activities. Staff and pupils alike suggested extending school hours or opening facilities in the afternoons as a cost-free way to provide healthy alternatives.

*“Not everyone can afford clubs… but if schools open in the afternoon for free, my mum would let me play there.” (Boy, boys’ school)*

Girls in particular emphasised the need for expanded access to books, art supplies, and creative spaces that aligned with their interests and offered a safe alternative to smoking.

*“Sometimes schools don’t have enough materials… you can’t even borrow books to read at home.” (Girl, girls’ school)*

In summary, strengthening automatic motivation requires a shift from punitive approaches to those that reinforce and normalise healthy behaviours. Combining consistent reward systems with accessible, enjoyable extracurricular options can proactively shape pupils’ emotional responses, habits, and social environments, creating positive associations with non-smoking and reducing the pull of tobacco.

## Discussion

This study explored strategies to strengthen the role of Egyptian preparatory schools in preventing adolescent smoking. Framed by the COM-B model, the findings highlight how schools can support behaviour change by enhancing pupils’ psychological capability, shaping physical and psychological opportunities, and influencing both reflective and automatic motivation. The study adds new contextual evidence from a conservative, resource-limited LMIC setting.

### Enhancing psychological capability: relevant knowledge and skill building

Improving adolescents’ knowledge and life skills is foundational for smoking prevention. Consistent with prior research, pupils in this study were more influenced by consequences that affect their personal and social lives than by distant health risks (68–71). Education must therefore go beyond generic health warnings and include the immediate consequences of smoking, such as appearance, physical fitness, sexual health, and peer relationships. Gender-sensitive messaging is vital as females responds more to content on beauty and relationships, while males engaged more with physical strength and self-image. This pattern is consistent with findings from other LMIC contexts (72–76).

Tailored content about different tobacco products (e.g., flavoured cigarettes, e-cigarettes, waterpipes) should be integrated into the curriculum and supported by visual aids, extracurricular sessions, and accessible posters. To further build psychological capability, pupils require life-skills training in refusal, stress management, decision-making, and assertiveness, skills that are largely missing in current curricula (37, 77–82).

### Reducing social and physical opportunities: role modelling and policy enforcement

Schools can limit opportunities for smoking by creating supportive environments and enforcing rules consistently. Banning smoking on school premises, including among staff, is crucial. When teachers or staff smoke, they model the behaviour for pupils, eroding credibility and weakening prevention efforts (77, 83, 84).

Participants called for staff sanctions, support to quit, and greater awareness of the impact of second-hand smoke. These strategies align with the Theoretical Domains Framework, which emphasises reducing environmental resources that enable risky behaviours (85). Promoting non-smoking role models—through awards, public recognition, and personal storytelling, was seen as a way to reinforce pro-health norms, a strategy also supported by findings from a systematic review of school-based interventions in LMICs.

Peer influence plays a central role in shaping adolescent behaviour (37, 45, 81). Informal peer education, peer-led discussions, and supportive social networks can foster collective resistance to smoking, an approach supported by evidence from both high- and low-income countries (37, 45, 86). Pupils who support non-smoking should be encouraged and recognised, fostering a culture where not smoking is socially valued which was effective in LMICs context (87–89).

Encouraging and recognising pupils who advocate for non-smoking was strongly recommended for helping to foster a culture in which abstaining from smoking is socially valued and is a strategy shown to be effective in LMIC contexts (45, 90, 91).

### Strengthening reflective motivation: persuasion and conscious decision-making

To support reflective motivation, pupils need opportunities to weigh the pros and cons of smoking and to critically evaluate their beliefs. This is supported by evidence from the Health Belief Model and Behaviour Change Wheel, which emphasise engaging both reason and emotion to support behaviour change (65, 83, 84). Traditional lectures were seen as less effective; pupils preferred persuasive, emotionally engaging methods such as storytelling, real-life case studies, school plays, and visual demonstrations.

Religion and family reputation were especially important for girls. Creating an expectation of parental disapproval or social shame around smoking strengthened pupils’ resolve to avoid it, particularly in a collectivist culture where group opinion carries weight in Egypt and countries with similar context (72, 92). Open discussions led by trusted teachers also recognised in supporting pupils in developing confidence in their non-smoking decisions.

### Enhancing automatic motivation: rewards and healthy alternatives

Adolescents’ behaviours are also shaped by emotional associations, rewards, and habits. While disciplinary measures have a place, participants emphasised the greater power of positive reinforcement, such as financial or social rewards for pupils who remain smoke-free or who support others. The Incentive Theory, the COM-B framework, and evidence from adolescent smoking prevention all support this approach, recognising the motivational power of anticipated rewards and highlighting its effectiveness (65).

Equally important is offering healthy behavioural alternatives. Pupils often smoke to cope with boredom, stress, or social exclusion (93). Encouraging hobbies and sports, making those accessible during or after school, can displace smoking-related habits (45, 94). However, lack of school resources and affordable extracurricular opportunities remain major barriers in Egypt and other LMICs (45, 86, 95). Girls in particular reported limited access to leisure activities, suggesting the need for gender-sensitive planning. This challenge is commonly observed in conservative communities and across many LMICs (86, 96–99).

These strategies align with the Theory of Planned Behaviour (100), which emphasises the importance of facilitating positive behaviours while restricting access to harmful ones. Participation in sports, in particular, was perceived to enhance self-confidence, reduce stress, and offer a smoke-free social environment; an approach supported by evidence from several effective school-based smoking prevention interventions (101).

### Barriers and enablers in low-resource, conservative settings

Although promising strategies were identified, several barriers to implementation were noted. These include under-resourced schools, a lack of trained staff, limited access to educational materials, and cultural taboos around discussing topics like smoking and sexual health. In line with previous LMIC studies (101–103), successful implementation of SBSPIs requires local adaptation, low-cost delivery models, and community engagement (37, 45, 82, 96, 98, 101).

### Methodological limitations

This study was conducted in three preparatory schools in Upper Egypt, which may limit the transferability of findings to other Egyptian regions or school types. Although schools were purposively stratified by gender composition to maximise diversity, selection relied on local gatekeepers, which may have introduced recruitment bias despite efforts to include staff and pupils of different ages, backgrounds, and smoking exposures. All interviews and FGDs were held within school premises, which could have influenced pupils’ openness to discuss sensitive topics, though privacy measures were implemented. Another limitation is the absence of parents’ perspectives, which were excluded to avoid potential harm or bias but could have enriched the analysis of school–family roles in prevention. Finally, this study did not examine in depth the capacity of schools to restrict students’ access to tobacco products outside school grounds, which remains an important area for future research.

## Conclusion

This study demonstrates that preparatory schools in conservative, resource-limited LMICs like Egypt provide an optimal location to implement targeted interventions which can shape future adolescent smoking prevention policies. Using the COM-B model, findings highlight the need to build pupils’ psychological capability through relevant education and life-skills training, reduce opportunities to smoke by reshaping school environments and norms, and influence motivation through persuasive communication, social reinforcement, and healthy alternatives. A whole-school approach, supported by trained staff, peer influence, and family engagement, is essential. Successful implementation depends on addressing contextual barriers and ensuring strategies are culturally sensitive, age-appropriate, and gender-responsive. These findings offer a practical framework to inform school-based smoking prevention efforts across similar low-resource settings.

### Implications for practice, policy, and future interventions

This study highlights the potential of preparatory schools in Egypt, and similar LMIC settings, to serve as a cornerstone for adolescent smoking prevention. For school-based interventions to be effective, they must address multiple behavioural drivers simultaneously.

Educational strategies should deliver age-appropriate, culturally relevant content on the immediate and long-term consequences of all tobacco forms, including waterpipes and e-cigarettes. This should be complemented by the integration of life-skills training, particularly in refusal, stress management, and decision-making, into the school environment.

Creating and maintaining smoke-free school settings is vital. This includes enforcing no-smoking school policies, providing cessation support for staff and smoking parents, and actively promoting non-smoking role models among both pupils and educators. Informal peer-led education and recognition schemes can further strengthen anti-smoking norms at schools.

To influence motivation, emotionally resonant approaches such as storytelling, visual demonstrations, and interactive activities should replace traditional lectures. Framing smoking as socially and morally unacceptable, especially through the lens of family and religious values, may be particularly persuasive for conservative and female pupils.

Incentives and structured extracurricular alternatives (e.g., sports, arts, reading clubs) can help displace smoking-related behaviours and meet pupils’ needs for stress relief and social belonging. Ensuring access to such opportunities, especially for girls, requires gender-sensitive planning and adequate school resources.

Finally, engaging parents, religious figures, and community leaders will be essential to reinforce smoke-free norms beyond school boundaries. Overcoming barriers such as staff shortages, limited resources, and cultural sensitivities is crucial for the long-term success and scalability of SBSPIs in fragile contexts.

For adolescent participants, a two-stage opt-in process was followed: parental/guardian consent was obtained first, followed by the pupil’s own written assent. Participation was voluntary, and pupils were included only when both pupil and parent/guardian consent were secured. Verbal consent was audio-recorded and witnessed for two staff participants who declined written consent due to cultural norms. All procedures followed institutional and international ethical guidelines for research involving human subjects and children in LMICs (104–113).

## Data Availability

The original contributions presented in the study are included in the article/supplementary material, further inquiries can be directed to the corresponding author.
